# Socioeconomic and environmental factors associated with waterpipe tobacco smoking among Iranian adults: a PERSIAN cohort-based cross-sectional study

**DOI:** 10.1186/s12889-023-16176-8

**Published:** 2023-07-05

**Authors:** Telma Zahirian Moghadam, Hamed Zandian, Mehdi Fazlzadeh, Mohammad Ebrahimi Kalan, Farhad Pourfarzi

**Affiliations:** 1 Social Determinants of Health Research Center, Ardabil University of Medical Sciences, Ardabil, Iran; 2 Centre for Public Health and Wellbeing, School of Health and Social Wellbeing, College of Health, Science and Society, University of the West of England, Bristol, UK; 3 Lung Diseases Research Center, Ardabil University of Medical Sciences, Ardabil, Iran; 4 School of Health Professions, Eastern Virginia Medical School, Norfolk, VA USA; 5 Digestive Disease Research Center, Ardabil University of Medical Sciences, Ardabil, Iran

**Keywords:** Tobacco, Non-smoked, Prevalence, Smoking, Waterpipe, Environment, Socioeconomic factors, Health status disparities

## Abstract

**Background:**

Waterpipe tobacco smoking (WTS) is associated with several deleterious health outcomes. We sought to estimate the prevalence of WTS and explore socioeconomic inequalities associated with this culturally-rooted tobacco smoking practice among Iranian adults.

**Methods:**

A cross-sectional analysis was conducted among 20,460 adults (ages 18 and older) enrolled in the PERSIAN cohort study during 2020. Data were collected on socioeconomic status (SES), lifestyle, alcohol consumption, cigarette smoking, and several risk factors related to non-communicable diseases. The concentration curve and relative concentration index (RCI) were administered to assess and quantify the SES-based inequality in WTS.

**Results:**

Overall age-adjusted prevalence of past-month WTS was 5.1% (95%CI:4.6–5.8), with about 1% for women and 10.6 for men. Age-adjusted prevalence of WTS was higher among younger adults, men, cigarette smokers, obese adults, and those with higher SES. The RCI estimation showed that WTS is more popular among adults with high income and education. WTS was higher among younger adults, cigarette smokers, obese adults, and those with higher SES.

**Conclusion:**

There is a clear socioeconomic inequality in WTS, with a higher prevalence among adults with higher income and education. The findings suggest the need for targeted interventions to address this inequality and reduce the prevalence of WTS among high-income communities.

## Background

**Fig. 1:**
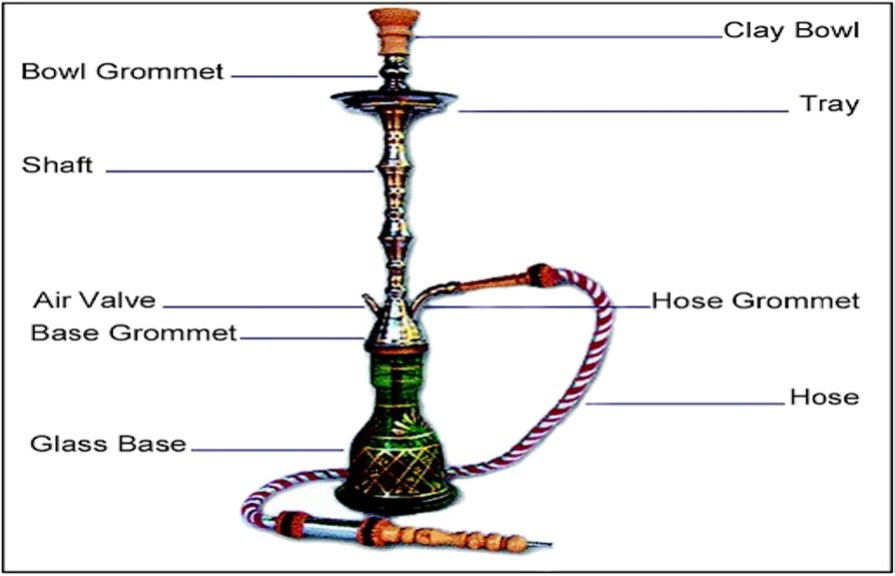
Schematic image of typical waterpipe tobacco smoking device, retrieved from Alqahtani et al., [14]

Tobacco smoking causes more than 8 million worldwide deaths annually [1]. One contributor to this burden is waterpipe tobacco smoking (WTS), also known as nargile, shisha, hookah, or galyan. WTS is a centuries-old smoking practice rooted in Eastern Mediterranean culture. It gained popularity worldwide since the 1990s after the introduction of flavoured tobacco known as Muʽassel (Arabic: معسل, meaning 'honeyed') (Massal) [2–4]. A typical WTS session lasts about an hour, accompanied by friends in a café or lounge [5–7], and is now the second most common form of tobacco consumption in Iran and neighbouring Arab countries [6, 8]. During a WTS session (as illustrated in Fig. 1), charcoal-heated flavoured or non-flavoured tobacco passes through a water-filled glass base, which cools the smoke before delivering nicotine and other toxicants into the user's lungs [9–12]. The type of tobacco (flavoured or non-flavoured), configuration, size, and appearance of the device varies from region to region [13].

The combustion produced during WTS session puts the smokers at detrimental health risks such as lung and cardiovascular diseases and many other dire health consequences [15, 16]. Despite the well-documented harmful effects of WTS on human health, many smokers believe that WTS is not as harmful and addictive as cigarettes due to water filtration, enticing flavors, and intermittent use, which are the main reason for popularity of WTS worldwide [17, 18].

Iran is one of the countries where WTS has a historical origin, and in recent years, the pattern of WTS has changed, being a more popular method of tobacco use among young adults, with increasing trends in women [6, 19–21]. The prevalence of current (past 30 days) WTS among Iranian adults ranges from 17 to 28% in different regions of Iran. Waterpipe use prevalence in men is significantly more than women's (24.2% vs. 11.3%) [6]. Ineffective enforcement of regulations regarding WTS and inadequate supervision of tobacco production(import and export), as well as scarce control of WTS in hookah bars contribute significantly to the growing popularity of WTS in Iran [6]. Monitoring tobacco use at population level-especially in low- and middle-income nations like Iran is one of the main goals for the World Health Organization Framework on Tobacco Convention Treaty (WHO FTCT) [22, 23]. Therefore, understanding the current condition and main factors that contribute to the popularity of WTS in developing countries is one of the critical factors to decrease the prevalence of WTS and eliminate waterpipe-induced addiction and its related health risks [24].

To better monitor the trend of WTS in Iran and help design targeted prevention and control programs, in this study we aim to evaluate the prevalence of WTS and related socio-economic and environmental factors in a large sample of Iranian adults participated at baseline wave of Prospective Epidemiological Research Studies in IrAN (PERSIAN) cohort study.

## Methods

### Study setting and sample

The research for this cross-sectional study was carried out in Ardabil, a city located in the northwest of Iran that has a population of about 610,000 individuals [25]. The study draws on data collected from the PERSIAN cohort study, which was established to modify healthcare policies related to non-communicable diseases (NCDs) and involved a variety of sites across Iran. Among these sites, the Ardabil NCD (ArNCD) cohort study was one of 18 geographically distinct locations included in the PERSIAN cohort study. Further information on the sampling design and methodology can be found elsewhere [26].

The research involved individuals mostly belonging to Turk ethnicities. The PERSIAN cohort study's extensive protocol aimed to recruit participants for this study, resulting in 20,525 men and women aged 18 years and above residing in Ardabil, who participated in the baseline survey between May 2017 and February 2020. As defined in study protocol [26, 27], the alluded age range was chosen for three reasons to be eligible in participating PERSIAN cohort: i) individuals in this age group are more likely to have well-established behaviors and lifestyles, 2) they are active and energetic enough to participate in a study; and iii) they will, within a reasonable amount of time, incur the PERSIAN study’s primary outcomes of interest (ie. a number of deaths, by cause and incidence of major NCDs) (see Poustchi et al. 2017 [26] for more details). The present study only included individuals who were Iranian citizens and residing in Ardabil. Those with medical conditions such as deafness, blindness, palsy, mental disorders, intellectual disability, or acute psychiatric illnesses were excluded from the PERSIAN cohort. Trained interviewers conducted the cohort questionnaire. After accounting for missing data, the final sample size for this study was 20,460 individuals. Ethics approval for the current analysis was obtained from the ethics committees of the Ministry of Health and Medical Education in Iran, as well as from the medical universities of each study site, including Ardabil University of Medical Science.

### Measures

#### Outcome

The outcome variable of the study was defined as the current use of waterpipe smoking (WTS) in the past 30 days prior to the interview time [6]. Participants who reported using waterpipes in the past 30 days were considered current waterpipe smokers. The outcome variable was dichotomous, meaning it only had two categories: current and non-current waterpipe smokers. We did not include ex-smokers or never-smokers in our outcome variable definition..

### Covariates

Selected covariates included age (categorized from under 35 (18–35 years) to upper 65), sex (man/ woman), marital status (single/ married/divorced/ widow), education status (no formal education, primary, intermediate, secondary, or academic degree), BMI (underweight/ normal/ overweight/ obesity) as previously defined [28], cigarette smoking (Current smoker (those who smoked one or more cigarettes a day for at least a month or those who reported having quit smoking for less than one month)/ nonsmoker) and chronic underlying disease (hypertension and diabetes (yes/no). Data about the non-communicable diseases (Diabetes and Hypertension) was extracted based on the individuals’ self-declaration, clinical tests results, and requests to see their clinical records.

### Wealth index

Wealth index (WI) was calculated as socioeconomic status (SES) of the participant based on their self-reported wealth, and it was divided into five quintiles (from 1st quintile as poorest to 5th quintile as richest groups). The WI is a composite index composed of key asset ownership variables; it is used as a proxy indicator of household household's cumulative living standard. To construct the wealth index, we need all the indicators that allow us to understand the level of wealth of the household. To create the WI, the Principal Component Analysis (PCA) was used. Filmer and Pritchett [29] popularized PCA for estimating wealth levels using asset indicators to replace income or consumption data. Following Filmer and Pritchett, many other studies, especially in economics and public policy, have implemented and recommended the use of PCA for estimating wealth effects [30, 31]. A PCA is run with all the selected variables. For constructing the wealth index, the principal component (first factor) presents the household’s wealth [32, 33]. The PCA is a multivariate statistical technique used to reduce the number of variables in a data set into a smaller number of ‘dimensions’. In mathematical terms, PCA creates uncorrelated indices or components from an initial set of n correlated variables, where each component is a linear weighted combination of the initial variables. For example, from a set of variables *X*_1_ through to *X*_*n*_,

$${PC}_{1}={a}_{11}{X}_{1}+ {a}_{12}{X}_{2}+\dots +{a}_{1n}{X}_{n}$$

$${PC}_{m}={a}_{m1}{X}_{1}+ {a}_{m2}{X}_{2}+\dots +{a}_{mn}{X}_{n}$$

where *a*_*mn*_ represents the weight for the *m*_i_ principal component and the *n*_i_ variable [33].

To calculate the wealth index by using PCA in this study, assets (having freezer, washing machine, dishwasher, laptop or PC, and etc.) homeownership, number of books read per year, home area, number of trips abroad, number of domestic trip, having a car and its price was considered. All the yes/no variables recoded in binary variables (0 = no vs 1 = yes). The variables with more than one category recoded in improved ‘1’ or not improved ‘0’ (when possible). Recode in binary categories that clearly distinguish ‘wealthier’ from ‘poorer’. We include in the PCA all the variables (assets, housing etc.) that we think will be appropriate to explain the household's wealth.

### Statistical analysis

Data were analyzed using Stata software, version 14.0.0 (Stata Corp, College Station, TX, USA). The results are presented as non-waterpipe smokers and current waterpipe smokers for the purpose of this analysis, separately. The categorical variables with corresponding 95% confidence intervals (95%CI) are presented as proportions. For the numerical variables, we calculated the means with the standard deviation (SD) and median with interquartile ranges. In order to achieve the crude and adjusted prevalence and their respective 95% CI, Poisson regression was used. Univariate and multivariable logistic regressions were fitted to our data accounting for demographic variables and chronic disease status. Crude and adjusted Odds Ratio (OR) and corresponding 95% CI were reported.

Relative Concentration Index (RCI) and Concentration Curve (CC) [34] examined SES differences in WTS among participants. The RCI was employed for quantifying and decomposing socioeconomic inequality in WTS among Ardabil adults. The concentration curve was used to calculate RCI, which diagrams the cumulative percentage of SES-ranked participants on the x-axis and the cumulative percentage of a health interest variable (i.e., WTS) on the y-axis. The RCI is equivalent to twice the area between the perfect equality line (45-degree line) and the concentration curve [35]. RCI values ranges from − 1 to + 1; with positive when the concentration curve lies below the line of perfect equality, and negative when this curve lies below the line. The RCI's positive (and negative) values indicate that the WTS concentrated more among the poorest (richest) [34]. The RCI was separated by $\frac{1}{1-\mu }$ to normalization following Wagstaff [36]. For this calculation, μ is assumed to be the measure of WTS. The decomposition process was used to classify the key determinants of the reported inequities of WTS [37]. There is a relationship regarding the WTS and other factors, ${x}_{k}$ [38].

$$y=\alpha +\sum_{k}{\beta }_{k}{x}_{k}+\varepsilon$$

where ${x}_{k}$ describes the explanatory variables alluded to in the previous portion. The RCI for WTS has been decomposed [39]:

$$RC=\sum_{k}\left(\frac{{\beta }_{k}{\overline{x} }_{k}}{\mu }\right){RC}_{k}+\frac{{AC}_{\varepsilon }}{\mu }$$

where $RC$ is the relative concentration index for WTS, ${\overline{x} }_{k}$ the mean of ${x}_{k}$ determinants, ${C}_{k}$ are the $RC$ for explanatory variables, and ${x}_{k}\left(\frac{{\beta }_{k}{\overline{x} }_{k}}{\mu }\right){RC}_{k}$ is the elasticity of WTS in relation to the explanatory variable ${x}_{k}$. $\sum_{k}\left(\frac{{\beta }_{k}{\overline{x} }_{k}}{\mu }\right){RC}_{k}$ presents the contribution of the explanatory factor ${x}_{k}$ to the$RC$. The last term,$\frac{{AC}_{\varepsilon }}{\mu }$, is the residual component.

## Results

Out of 20,460 participants, 45.8% were men, mostly 46–55 years (35.1%). The mean age of participants was 49.0 (95% CI: 48.92 to 49.2) years of the total participants, 16.1% were cigarette smokers, more than 42% were obese, and 20.8% and 11.7% had hypertension and diabetes, respectively.

The overall prevalence of WTS was 5.14% (95% CI: 4.85 to 5.45), with an overall age-adjusted prevalence of 5.12% (95% CI: 4.63 to 5.79). The prevalence among women was 0.72% (95% CI: 0.57 to 0.88), while among men, it was 10.64% (95% CI: 10.1 to 11.2). The prevalence of WTS was higher among adults who were married (5.5%), had primary school level education (5.69%), smokers (13.95%), had normal BMI(5.82%), and richest group (8.87%) (Table 1).

**Table 1:** Baseline characteristics and prevalence of WTS in ArNCD cohort study (*n* = 20,460)

	**Overall n (%)**	**Prevalence of WTSs**	
		Crude (95% CI)	Age-adjusted (95% CI)
**Age group**			
< 35	398 (1.95)	8.29 (5.9 to 11.4)	1.61 (1.11 to 2.10)^*^
35–45	6815 (33.3)	6.54 (5.9 to 7.2)	2.17 (1.99 to 2.36)^*^
46–55	7593 (37.1)	4.13 (3.70 to 4.60)	1.53 (1.37 to 1.69)^*^
56–65	4597 (22.5)	4.28 (3.73 to 4.91)	0.96 (0.83 to 1.09)
> 66 years	1057 (5.2)	5.96 (4.68 to 7.56)	0.31 (0.23 to 0.37)^*^
**Sex**			
Men	9377 (45.83)	10.33 (9.73 to 10.96)	10.64 (10.01 to 11.2)^*^
Women	11,083 (54.17)	0.75 (0.61 to 0.93)	0.72 (0.57 to 0.88)^*^
**Marital status**			
Single	341 (1.67)	4.10 (2.43 to 6.82)	1.57 (0.53 to 2.61)
Married	18,611 (90.9)	5.49 (5.17 to 5.82)	5.50 (5.18 to 5.83)^*^
Divorced/Widowed	1508 (7.4)	1.12 (0.70 to 1.80)	1.70 (0.84 to 2.57)
**Years of schooling**			
No formal education	6530 (31.9)	2.48 (2.13 to 2.88)	2.81 (2.23 to 3.38)^*^
Primary (1–5 years)	4605 (22.5)	5.21 (4.60 to 5.89)	5.69 (4.97 to 6.41)^*^
Intermediate (6–9 years)	3061 (14.9)	6.66 (5.83 to 7.60)	6.52 (5.55 to 7.50)^*^
Secondary (10–12 years)	3460 (16.9)	6.79 (5.99 to 7.68)	6.74 (5.83 to 7.64)^*^
Academic (13 and above)	2804 (13.7)	7.56 (6.63 to 8.59)	6.95 (5.99 to 7.91)^*^
**Smoking status**			
Smoker	3291 (16.0)	12.82 (11.7 to 14.0)	13.95 (12.7 to 15.2)^*^
Non-smoker	17,169 (83.9)	3.67 (3.40 to 3.96)	3.62 (3.34 to 3.90)^*^
**BMI**			
Normal weight	3304 (16.2)	6.02 (5.26 to 6.88)	5.82 (5.03 to 6.62)^*^
Over weight	8470 (41.4)	5.73 (5.26 to 6.25)	5.64 (5.15 to 6.13)^*^
Obesity	8686 (42.5)	4.23 (3.83 to 4.68)	4.46 (4.01 to 4.90)^*^
**Hypertension**			
Yes	4247 (20.8)	3.50 (2.99 to 4.10)	5.46 (12.8 to 14.1)^*^
No	16,213 (79.2)	5.57 (5.23 to 5.93)	3.82 (2.92 to 4.72)^*^
**Diabetes**			
Yes	2387 (11.7)	4.57 (2.15 to 6.19)	5.01 (3.08 to 8.61)^*^
No	18,073 (88.3)	5.22 (3.23 to 7.82)	4.20 (2.02 to 6.11)^*^
**Cardiovascular Diseases**			
Yes	1739 (8.5)	3.01 (1.41 to 7.82)	5.42 (2.88 to 7.94)^*^
No	18,721 (91.5)	8.18 (4.13 to 11.9)	3.66 (1.59 to 7.02)^*^
**Socio-economic status**			
Poorest	4092 (20.0)	2.85 (2.39 to 3.41)	2.93 (2.40 to 3.47)^*^
Poor	4092 (20.0)	3.29 (2.79 to 3.89)	3.30 (2.75 to 3.86)^*^
Middle	4092 (20.0)	4.25 (3.67 to 4.91)	4.22 (3.61 to 4.83)^*^
Rich	4102 (20.1)	6.19 (5.49 to 6.97)	6.19 (5.45 to 6.93)^*^
Richest	4082 (19.9)	9.13 (8.29 to 10.0)	8.87 (7.99 to 9.76)^*^

*WTS* Waterpipe toibacco smoking, *ArNCD* Ardabil non-comminicable diseases, *, significant at *p* < 0.05

As presented in Table 2, WTS prevalence decreased among older adults compared to younger adults. Men were more likely to be current waterpipe smokers compared to women (12.9, 95% Ci:10.10–16.49). Adults with primary education were 1.3-fold more likely to be waterpipe smokers than their no formal education counterparts. However, with the increase in the level of education, the relationship between education level and WTS yielded null results. Cigarette smokers were 74% more likely to be waterpipe smokers than those who were not cigarette smokers. The richest people were about 210% more likely to be waterpipe smokers than the poorest people.

**Table 2:** Association between explanatory variables and the prevalence of WTS (logistic regression model)

	**Odds ratio**		
	Crude (95% CI)	Adjusted (95% CI)	*p*-value
**Age group**			
< 35 (ref.)	1	1	1
35–45	0.77 (0.53 to 1.12)	0.71 (0.48 to 1.05)	0.093
46–55	0.44 (0.32 to 0.69)^*^	0.34 (0.22 to 0.51)	< 0.001
56–65	0.49 (0.33 to 0.72)^*^	0.38 (0.25 to 0.57)	< 0.001
≥ 66 years	0.70 (0.45 to 1.08)^*^	0.53 (0.33 to 0.86)	0.010
**Sex**			
Weman (ref.)	1	1	1
Men	15.09 (12.05 to 18.89)^*^	12.90 (10.10 to 16.49)	< 0.001
**Marital status**			
Single (ref.)	1	1	1
Married	1.35 (0.79 to 2.32)	1.01 (0.56 to 1.79)	0.970
Divorced/Widowed	0.26 (0.12 to 0.54)^*^	1.07 (0.50 to 2.30)	0.853
**Years of schooling**			
No formal education (ref.)	1	1	1
Primary (1–5 years)	2.16 (1.76 to 2.64)^*^	1.29 (1.04 to 1.61)	0.019
Intermediate (6–9 years)	2.80 (2.27 to 3.46)^*^	1.17 (0.92 to 1.48)	0.199
Secondary (10–12 years)	2.86 (2.33 to 3.51)^*^	1.08 (0.84 to 1.38)	0.530
Academic (13 and above)	3.21 (2.60 to 3.96)^*^	0.85 (0.65 to 1.12)	0.261
**Smoking status**			
Non-smoker (ref.)	1	1	1
Smoker	3.85 (3.38 to 4.38)^*^	1.74 (1.52 to 2.00)	< 0.001
**BMI**			
Normal weight (ref.)	1	1	1
Over weight	0.94 (0.80 to 1.12)	1.14 (0.96 to 1.37)	0.121
Obesity	0.69 (0.57 to 0.82)^*^	1.47 (1.21 to 1.78)	< 0.001
**Hypertension**			
No (ref.)	1	1	1
Yes	0.61 (0.51 to 0.73)^*^	0.89 (0.73 to 1.09)	0.291
**Diabetes**			
No (ref.)	1	1	1
Yes	0.86 (0.70 to 1.06)	1.14 (0.91 to 1.42)	0.232
**Cardiovascular Diseases**			
No (ref.)	1	1	1
Yes	1.03 (0.59 to 2.27)	1.29 (0.86 to 2.68)	0.069
**Socio-economic status**			
Poorest (ref.)	1	1	1
Poor	1.15 (0.90 to 1.49)	1.05 (0.81 to 1.37)	0.663
Middle	1.50 (1.18 to 1.91)^*^	1.20 (0.93 to 1.55)	0.142
Rich	2.24 (1.79 to 2.80)^*^	1.56 (1.22 to 1.99)	< 0.001
Richest	3.41 (2.76 to 4.22)^*^	2.11 (1.63 to 2.73)	< 0.001

Asterisks (*) indicate *p* < 0.05 unadjusted (crude) ORs

**Fig. 2:**
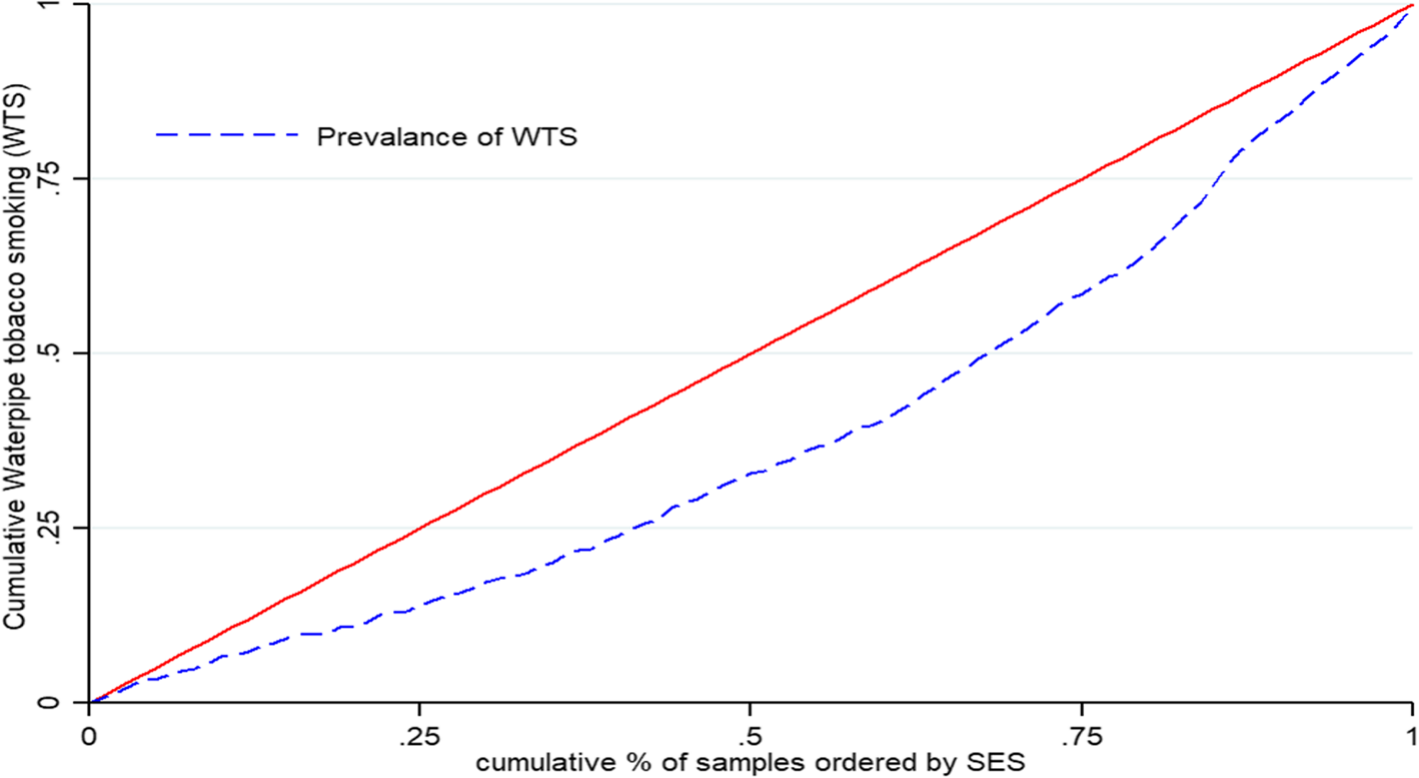
Concentration curve (CC) prevalence of WTS among adults in ArNCD cohort, where the prevalence of WTS is pro-rich and unequally distributed. The CC of WTS is positive and pro-rich, which indicates the significant inequality in WTS is towards the rich group in the population

**Fig. 3:**
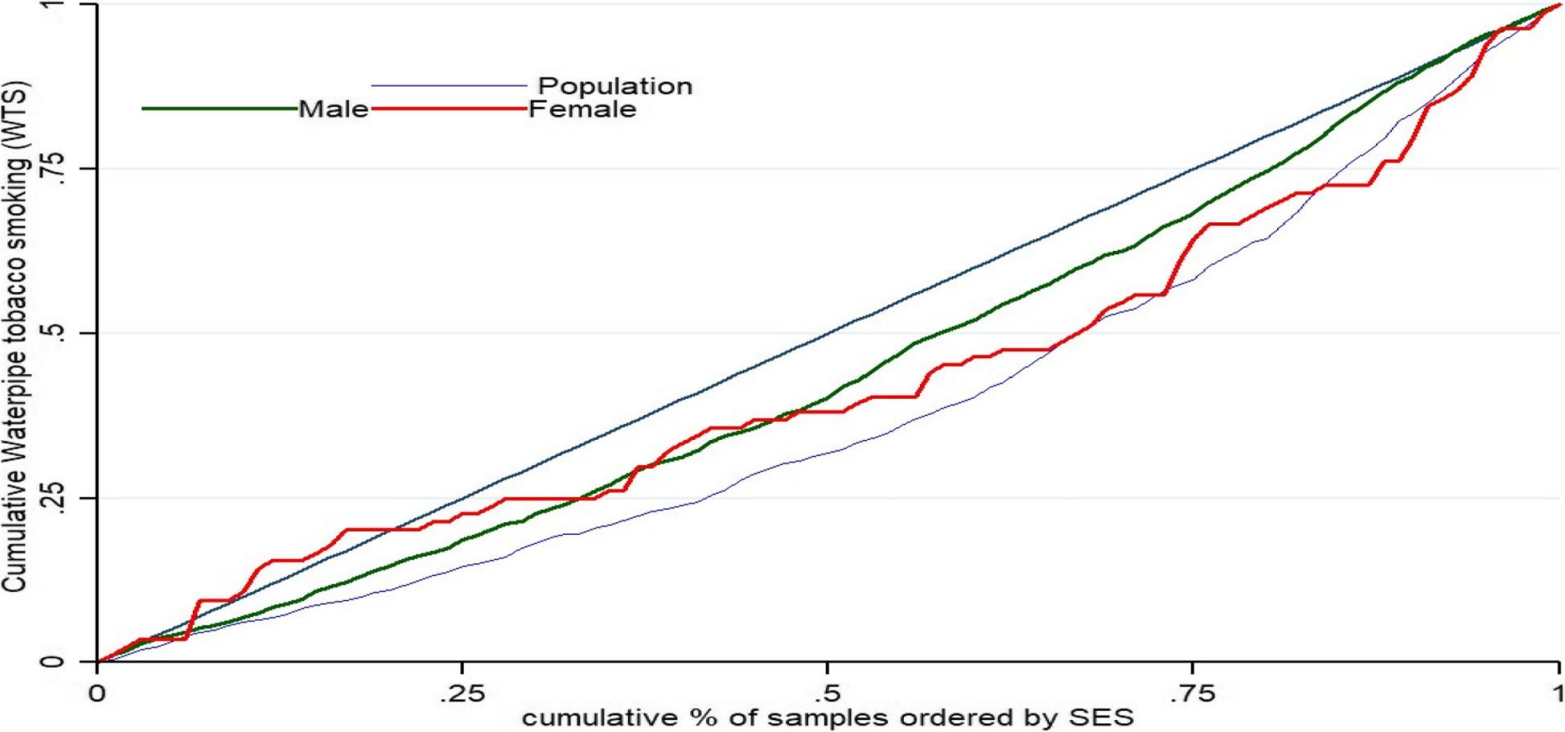
Concentration curve (CC) prevalence of WTS among adults in ArNCD cohort, separately for men and women. Among men, CC of WTS was positive and significantly pro-rich, but among women. No difference was observed between rich and poor among women in terms of WTS

**Fig. 4:**
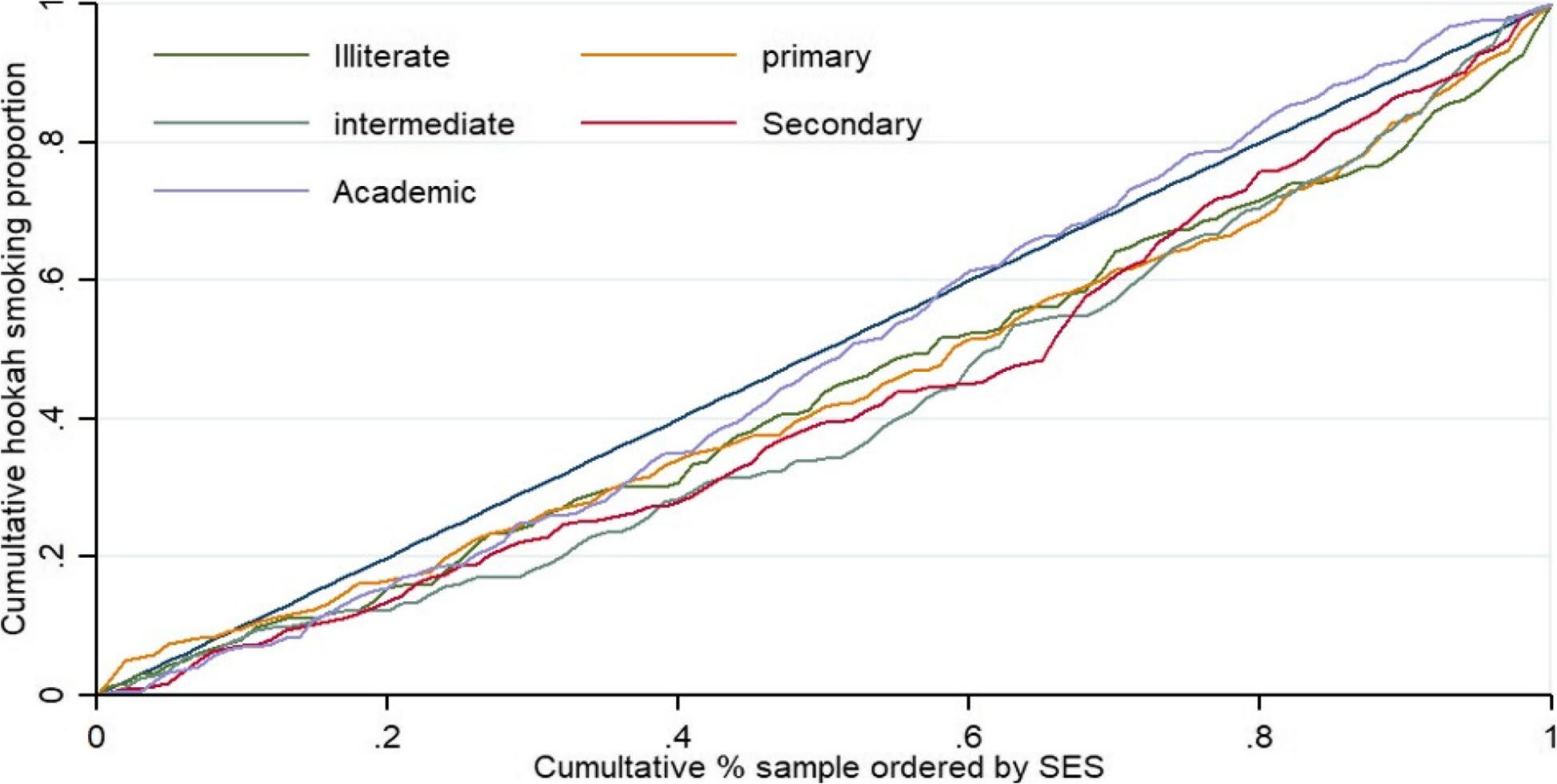
Concentration curve (CC) prevalence of WTS among adults in ArNCD cohort stratified by education level. Distribution of WTS did not differ significantly between rich and poor groups among people with no education, primary and academic education level. There was however significant pro-rich inequality in WTS among those with intermediate and secondary level education

Table 3 illustrates the estimated concentration index (CI), where CI was 0.25 (95% CI: 0.23 to 0.27) for all participants. A positive CI value means WTS is at a higher rate among the richest group (Fig. 2). There was a positive value for CI for men (0.13;95% CI: 0.111 to 0.149) and women (0.163;95% CI: 0.102 to 0.224), in terms of WTS inequality, in both cases gender WTS were in favor of richest group. In addition, estimated inequality for WTS among education level was in favor of the richest group, with positive CI in all education levels. It was 0.150 for no formal education and 0.081 for the population with an academic degree. Figures 3 and 4 display the concentration curve (CC) for inequality in WTS among participants based on gender and educational level, respectively.

**Table 3:** Concentration index for WTS based on socioeconomic status, education level, and sex

	Index Value	S.E	*P*-value	95% CI	
				**LB**	**U**
**CI for WTS for SES**	0.253	0.017	< 0.001	0.236	0.271
**CI for WTS for Education level**					
No formal education	0.150	0.043	< 0.001	0.107	0.196
Primary (1–5 years)	0.131	0.037	0.004	0.094	0.168
Intermediate (6–9 years)	0.196	0.040	< 0.001	0.156	0.236
Secondary (10–12 years)	0.181	0.037	< 0.001	0.144	0.218
Academic (13 and above)	0.081	0.034	0.018	0.047	0.115
**CI for WTS for male and female**					
Men	0.130	0.019	< 0.001	0.111	0.149
Women	0.163	0.061	0.008	0.102	0.224

## Discussion

This large sample cross-sectional study from a developing country highlights a comprehensive picture of the WTS in the within the context of socioeconomic inequality, which is not fully addressed in previous studies. Socioeconomic disparities in WTS were found to be more pronounced among affluent adults with higher educational attainment compared to those who were poor and had no formal education. The overall age-adjusted prevalence of WTS was 5.12% and it was 12 times more common among men than women. The prevalence of current WTS among the study participants was lower than most studies in Iran (8.6%-43.8%), which is likely related to the target population since in most studies, young people over 18 years old were considered [8, 19, 40], but in our study, about adults over 35 years old were included in the study because of their SES stability. However, the influence of SES on smoking patterns among this age group and the impact of SES instability remains unclear [20, 41], despite several studies indicating a higher prevalence of WTS among individuals aged 18–24 years with university education. Therefore, understanding this gap in older adults can add necessary information to growing literature and benefit targeted WTS intervention cessation programs.

The most important finding of this study is that inequality in WTS that was more pronounced for pro-rich in Iran, where it is in contrast to cigarettes smoking that is more popular among the poorest group than the richest, as observed in the same cohort earlier [42]. Previous studies, using the same methodology, found inequality in expenditure on tobacco, primarily cigarettes (as a share of household budget) disproportionality concentrated among poorer households in Iran [43]. This discrepancy highlights the popularity of cigarettes among people with lower SES, which could be different from WTS that is historically popular among people with higher SES in Middle Eastern culture [44]. Although about a decade ago, an Iranian study showed a lower prevalence of WTS in the high SES group than low SES [20], it is crucial to monitor tobacco use prevalence with a large sample to develop effective measures to limit its popularity [45]. A few studies in Iran are not in line with our results, where Ghelichkhani et al. found that the prevalence of WTS was lower in the group with high SES than in the group with low SES, and the gap was significant between the two mentioned SES groups [46]. One possible reason for the difference between the results of the present study and Ghelichkhani et al. is that our study focused on Iranian adults aged over 35 years who had achieved relative SES stability. In contrast, Ghelichkhani et al. studied individuals aged 7 to 70 years, which may have diluted the impact of SES status on WTS. Relatedly, the preparation of WTS or gathering in groups of friends at hookah café requires time and may cost more for people from a low-income neighborhood, contrary to cigarettes that are more easily accessible and cheaper for this low SES group [47]. In contrast to the findings of the present study, a study of Iranian adults in 2010 suggested that income had no significant effect on hookah use [48]. It should be noted that SES conditions have changed in recent years, and WTS on its own is much more expensive than cigarette smoking in Iran. The median and interquartile range of the monthly cost of WTS in Iran was estimated to be about US$3 (about 90,000 IRR) and US$15 (about 4,500,000 IRR), respectively, which is almost the same as a pack of cigarettes [6]. Furthermore, the depreciation of the Iranian currency exacerbates this burden, making it increasingly challenging for individuals with lower socioeconomic status to access and enjoy the welfare-to-service benefits, especially when compared to their wealthier counterparts. This could be another important reason why the results of this study are not in line with some previous studies that were conducted during a time, when Rial (Iranian currency) was enjoying its high value and WTS may have been more affordable for lower SES as high SES. Finally, it should be noted that hookah often is offered in expensive and luxury cafes in Iran and is considered a kind of luxury entertainment [49].

Studies conducted beyond the Middle East [50] have found that young people of higher SES are particularly at risk for WTS, highlighting the differences in SES levels between WTS and cigarette smoking. Earlier studies from Pakistan have shown that the highest rates of WTS were observed among college students with higher SES [51]. Danaei etal. in a cross-sectional study showed that the rate of WTS in the age group 18 to 24 years was 4.9 times higher than that in the group 45 years and older [6], which is in line with other studies in Iran [52]. However, this study reported similar results that young men, highSES, and urban groups are associated positively with WTS. One possible cause of this problem could be a lack of healthy entertainment options for young people in Iran and neighboring countries.

Similar to cigarette smoking, gender differences were observed among waterpipe smokers. Our study showed that men smoke waterpipe about 12 times more than women in north-west of Iran. This finding is consistent with studies conducted in other parts of Iran, Vietnam, Egypt, USA, and Turkey [19, 42, 53, 54]. In contrast, three studies conducted in Hormozgan [55] and Bushehr [56] and Kuwait [57] showed a higher prevalence of WTS among women than men. These differences are logical, as women's WTS in the eastern mediterranean region is traditional and rooted in culture. The tendency to WTS is seen among women in many societies, especially Middle Eastern nations, as it is believed that WTS is less dangerous and less stigmatized than cigarette smoking. Also, in Iranian society, some cultural and religious factors prevent women from smoking cigarettes [49].

According to our findings, there is a correlation between education level and socioeconomic (SES) inequality in regard to WTS, with a higher prevalence of WTS observed among individuals with higher education levels. A study conducted in Iran by Rezaei and colleagues demonstrated that the concentration of tobacco use among low-income households was primarily influenced by wealth and education [43]. A study in the US revealed that individuals with higher education were more likely to smoke waterpipe, and people living outside of poverty were disproportionately more likely to smoke waterpipe compared to whites [58].

The prevalence of tobacco use in a society is a helpful indicator of estimating its harmful health effects. Due to the risk factor of smoking, especially WTS, for many non-communicable diseases, if the current situation continues and no special intervention is taken, If the current situation continues without any special intervention, the inequality in WTS is likely to contribute to an unequal burden of WTS-induced diseases. Hypertension and diabetes as risk factors of heart diseases are associated significantly with higher odds of WTS in this study. Islami et al. showed that WTS was significantly associated with heart disease prevalence [59].

The prevalence of WTS in this study was lower than in other studies [6, 60], although the older age range is one of the main limitations of this study (see limitations section). WTS seems to be increasing in Ardabil. This is not only worrying but also shows a great deal of interest in WTS, which could be due to misconceptions about the safety of WTS. Abdollahifard et al. found that the popularity of WTS increased during the last decade and will increase over the next half-decade [19]. Therefore, monitoring WTS and how SES can affect pattern of WTS is crucial for prevention and cessation programs.

## Limitations

A limitation of this study is that, given the study's cross-sectional nature, the causal relationship between WTS and findings should be interpreted with caution. In addition, data were collected as a self-report, which could lead to reporting/response bias, for which a valid Persian cohort questionnaire was used and interviews. The study population consists of participants over 35 years of age since PERSIAN cohort’s primary purposes are collecting data to better understand the trajectories of NCDs outcomes.In order to generalize the results to the entire population of Iran, more comprehensive studies are necessary to include ages above 15, especially young adults who encompass a large proportion of waterpipe tobacco smokers in Iran. The strength of this research is the large sample of 20,427 people in the Ardabil Persian Cohort. A standard PERSIAN cohort questionnaire compares the results with other study results from different sites of Iran and future multicenter prospective studies from different sites of PERSIAN cohort would add synergy to growing literature about SES and how it can change over time.

## Conclusion

This study from a large sample of adults showed that rich people are more at risk of WTS than poor people, showing WTS as a pro-rich mode of smoking in the northwest of Iran. The index and concentration curve measures showed the existence of SES inequalities in WTS in the northwestern region of Iran. Waterpipe-specific prevention and interventions (e.g., high taxation and intensify monitoring of waterpipe supply centers) are needed to implement policies to reduce tobacco use among adults. Future similar studies are warranted to explore the SES inequlities in WTS among adults aged < 35, especially youths considering instability in their SES and matching it with parents or guardians SES.

## Data Availability

Data are available from the authors upon reasonable request to corresponding authors.

## Abbreviations

Ar-NCDs: Ardabil Non-Communicable Diseases
CC: Concentration Curve
NCDs: Non-communicable diseases
PCA: Principal Component Analysis
PERSIAN: Prospective Epidemiological Research Studies in IrAN
RCI: Relative Concentration Index
SES: Socioeconomic Status
